# Trends in prescription opioid use in Europe: A DARWIN EU^®^ multinational cohort study including seven European countries

**DOI:** 10.3389/fphar.2025.1608051

**Published:** 2025-08-18

**Authors:** Junqing Xie, Mike Du, Yuchen Guo, Cesar Barboza, James T. Brash, Antonella Delmestri, Talita Duarte-Salles, Jasmine Gratton, Romain Griffier, Raivo Kolde, Wai Yi Man, Núria Mercadé-Besora, Marek Oja, Sarah Seager, Katia Verhamme, Dina Vojinovic, Edward Burn, Daniel Prieto-Alhambra, Martí Català, Annika M. Jödicke

**Affiliations:** ^1^ Pharmaco- and Device Epidemiology Group, Centre of Statistics in Medicine, NDORMS, University of Oxford, England, United Kingdom; ^2^ Department of Medical Informatics, Erasmus Medical Center, Rotterdam, Netherlands; ^3^ IQVIA, Real World Solution, Brighton, United Kingdom; ^4^ Fundació Institut Universitari per a la recerca a l’Atenció Primària de Salut Jordi Gol i Gurina (IDIAPJGol), Barcelona, Spain; ^5^ Public Health Department, Medical Information Service, Bordeaux University Hospital, Bordeaux, France; ^6^ Institute of Computer Science, University of Tartu, Tartu, Estonia; ^7^ IQVIA, Real World Solutions, Amsterdam, Netherlands

**Keywords:** opioids, trends multinational, electronic health records, real-world data, common data model

## Abstract

**Background:**

The opioid crisis has been a serious public health challenge in North America for decades, despite numerous efforts to mitigate its devastating consequences. As concerns grow about a similar situation developing in Europe, we evaluated the trends in opioid use and characterized prescribing indications across seven European countries.

**Methods:**

We conducted a multinational cohort study using electronic health records from various healthcare settings: primary care [Clinical Practice Research Datalink (CPRD) GOLD (United Kingdom), Sistema d’Informació per al Desenvolupament de la Investigació en Atenció Primària (SIDIAP, Spain), and Integrated Primary Care Information Project (IPCI, the Netherlands)]; primary and outpatient specialist care [IQVIA Disease Analyzer (DA) Germany and IQVIA Longitudinal Patient Database (LPD) Belgium]; hospital care [Clinical Data Warehouse of Bordeaux University Hospital (CHUBX, France)]; and the Estonian Biobank (EBB). All data were mapped to the Observational Medical Outcomes Partnership (OMOP) Common Data Model (CDM). All people registered in a contributing database for ≥365 days between 2012 and 2022 were included. Annual period prevalence and incidence rates of opioid prescriptions were estimated, and long-term trends were quantified as the percent change from 2012 to 2019. New opioid users were characterized, including potential prescribing indications.

**Results:**

Between 2012 and 2019, the incidence of opioid prescriptions in primary care decreased by −50·7% (CPRD GOLD) and −2·0% (SIDIAP), while it increased in EBB (+52·8%) and CHUBX (+25·3%) data. The incidence of codeine and tramadol use decreased in most databases. However, the prevalence of oxycodone, morphine, and fentanyl increased. Opioid use was highest among older age groups, and the majority of prescriptions were for oral formulations. Respiratory and pain-related conditions were the most common indications for new opioid users in outpatient settings.

**Conclusion:**

Despite a decrease in new opioid prescriptions in many European countries, the prevalence of opioid use remained largely stable over the last decade. More data are needed to monitor evolving opioid prescription patterns in Europe, particularly in the post-pandemic era.

## Research in context

### Evidence before this study

The opioid epidemic has persisted as a public health crisis in North America for decades, despite numerous efforts to mitigate its devastating consequences. Concerns about a similar situation evolving in Europe have led to increased monitoring, primarily focusing on illicit opioid use, dependence, and fatal overdose. Present studies often used different methodologies for measuring opioid use, thus limiting comparisons across countries and healthcare settings. Studies on the overall use of prescription opioids that can inform health policy and regulatory decision making in Europe are scarce.

### Added value of this study

Utilizing standardized methods, the multinational Data Analysis and Real-World Interrogation Network (DARWIN EU^®^) study assessed opioid use across various healthcare settings in Europe. Our study highlights that, despite a decrease in new opioid prescriptions in many European countries, the prevalence of opioid use has remained largely stable over the last decade. A higher prevalence was particularly observed among older people, along with increasing use of potent opioids, including oxycodone, morphine, and fentanyl, which are under particular monitoring. Treatment duration and indications varied greatly across settings.

### Implications of all the available evidence

The European Medicines Regulatory Network (EMRN) requested this study to support regulatory decision making and promote the safe use of opioids in Europe.

## Introduction

The opioid crisis has been a serious public health challenge for decades. Concerning rates of opioid dependence among young adults following opioid prescription and their association with overdoses and opioid-related deaths have been widely reported in the United States. In recent years, the number of opioid overdoses and related deaths has tripled in the United States, with the increase being primarily caused by fentanyl and its analogs (Gomes et al., 2023). Despite the implementation of numerous interventions and initiatives, rates of fentanyl-related fatalities are expected to remain high in the near future (Ballreich et al., 2020). Concerningly, emerging data suggest that the opioid crisis has already extended to other high-income countries, including Canada (Cheung et al., 2023; Vojtila et al., 2019), Australia (Australian Government: Department of Health and Aged Care, 2019; Roxburgh et al., 2017), and parts of the United Kingdom (Kimber et al., 2019; Alenezi et al., 2021).

Recent surveillance studies suggested that Europe is not experiencing a similar opioid crisis, despite a reported increase in opioid usage in many European countries (Hauser et al., 2021; van Amster et al., 2015; Seyler et al., 2021). Nevertheless, concerns remained as risk factors for prolonged use and development of dependence, such as chronic pain, mental health disorders, and advanced age, are common in the European population.

Those concerns should not be an obstacle to the improvement of pain management (Marchetti Calonego et al., 2020), given the high efficacy of opioid therapy for acute and cancer pain, along with its importance in palliative care (Paice et al., 2023). Close monitoring and surveillance of opioid utilization are increasingly important to detect early signs of opioid-related issues and allow for the implementation of preventative measures where needed. To our knowledge, no studies have systematically assessed opioid prescriptions across European countries and healthcare settings, particularly with regard to substance-specific prescription trends. Substantial heterogeneity in definitions of opioid use and research methodologies in previous studies hindered effective cross-country and regional comparisons (Kalkman et al., 2022; Karmali et al., 2020).

The European Medicines Regulatory Network (EMRN) requested this drug utilization study through the Data Analysis and Real-World Interrogation Network (DARWIN EU^®^) initiative to assess recent trends in opioid prescribing and characterize new opioid users and indications across different healthcare settings in Europe.

## Methods

### Data sources

This study was conducted using de-identified, routinely collected data from seven European countries: the United Kingdom, the Netherlands, France, Spain, Belgium, Germany, and Estonia. The databases were selected based on their availability as established data partners within the DARWIN EU^®^ at the time of the study. This network was designed to comprise a variety of European countries and healthcare settings, including primary care, specialist care, and hospital data, to facilitate the generation of robust multinational evidence for regulatory decision making.

Primary care electronic health records were retrieved from the UK’s Clinical Practice Research Datalink [CPRD GOLD] (Herrett et al., 2015), the Integrated Primary Care Information Project (IPCI) (de Ridder et al., 2022) from the Netherlands, and the Sistema d’Informació per al Desenvolupament de la Investigació en Atenció Primària (SIDIAP) (Recalde et al., 2022) from Catalonia, Spain. The IQVIA Longitudinal Patient Database Belgium (IQVIA LPD Belgium) and IQVIA Disease Analyzer Germany (IQVIA DA Germany) contributed electronic health records covering both primary care and outpatient specialist care practices. The Clinical Data Warehouse of Bordeaux University Hospital (CHUBX), France, contributed in- and outpatient data from hospital care, while the Estonian Biobank (EBB) (Leitsalu et al., 2015) contributed claims data. A detailed description of these databases is provided in Supplementary Table S1.

All seven databases were mapped to the Observational Medical Outcomes Partnership (OMOP) Common Data Model (CDM) to allow for federated standardized analytics in the context of the DARWIN EU^®^ initiative (link).

### Study participants

The study period was between 1 January 2012 and 31 December 2022. The source population included all people with at least 1 year of prior history available in the respective database. For incidence rate estimations and new user characterization, new opioid users were defined as people with a record of an opioid prescription who had not received the same opioid within the previous 12 months. Sensitivity analyses were conducted using a 6-month washout period instead.

### Exposure of interest

The exposure of interest was opioids, including all substances from the Anatomical Therapeutic Chemical (ATC) classes N01AH, N02A, and R05DA available in the respective countries. We grouped opioids as an overall substance class and stratified them by route of administration (oral, transdermal, or parenteral), potency according to the WHO Analgesic Ladder (Anekar et al., 2023) (weak or potent), and individual substance. All products containing the respective opioids were identified, including combinations with non-opioid medicines. A list of all opioids included in the study is provided in Supplementary Table S2.

In addition, a subset of opioids was selected as opioids of special interest for specific analysis, which consists of the weak opioids codeine and tramadol and the potent opioids hydromorphone, fentanyl, morphine, and oxycodone. They were selected because they are among the most commonly prescribed and have been highlighted in prior research for significant trends or safety concerns that warrant closer monitoring (Gomes et al., 2022; Gomes et al., 2018; Iaboni et al., 2019).

### Statistical analysis

#### Trend in opioid use over time: incidence and prevalence

Prevalence was calculated as annual period prevalence (PP), summarizing the total number of participants who used opioids during a given year, divided by the denominator population for that year. People in the source population contributed to the denominator from the study start date (e.g., 1 January 2012) until the earliest of the study end date (31 December 2022) or end of observation (i.e., date of data extraction, death, people leaving the general practitioner practice, or the practice stopping contributing data to the database). Therefore, period prevalence reflects the proportion of individuals exposed at any time during a specified interval. Incidence rates (IRs) were calculated as the number of new users per 100 person-years of the population at risk of exposure during each calendar year. Incidence rates and prevalence were stratified by age, using 10-year age bands. The percent change in opioid prescription rates was calculated from 2012 to the beginning of the COVID-19 pandemic (between 2012 and 2019) using the following formula: [(rate in 2019 − rate in 2012)/rate in 2012] × 100.

#### Patient-level opioid use

New opioid users were characterized by describing patient demographics and history of comorbidities and co-medication at the time of their first opioid prescription (index date). Potential indications were assessed by reviewing diagnoses recorded at the index date and in the week and month before as the indication was not explicitly recorded alongside the prescription in our databases. Treatment duration was estimated for the first treatment era and summarized using the median and interquartile range.

#### DARWIN EU network study

This study was carried out within the DARWIN EU^®^ framework, using standardized analyses. All analyses were pre-specified in a publicly available protocol (EUPAS Register No 105641) and then carried out in a federated manner, separately for each database. All databases were onboarded as data partners for the DARWIN EU^®^ initiative. The study code was written in R using the IncidencePrevalence (Raventós et al., 2023), PatientProfiles, and DrugUtilisation (Catala et al., 2025) packages. Database partners ran the analytical code locally, and only the summarized results were shared. A minimum cell count of five was used when reporting results to preserve data privacy, and any smaller counts were reported as <5.

The full study code is available on GitHub [darwin-eu-studies/P2-C1-002-OpioidsDrugUtilisation (github.com)] to maximize transparency and reproducibility.

### Role of the funding source

The study sponsor (EMA/EMRN) requested a drug utilization study for opioids. This study was funded by the EMA and performed via DARWIN EU^®^. The lead investigators (JX and AMJ) designed the study using the methods defined in the DARWIN EU^®^ Catalogue of Standard Analytics. The EMA reviewed and approved the study protocol. The EMA had no role in the collection, analysis, and interpretation of data. The full study report was reviewed and approved by the EMA, which also reviewed the manuscript prior to publication.

## Results

The results from all analyses are available in an interactive web application (https://dpa-pde-oxford.shinyapps.io/P2C1002OpioidStudy/).

### Study population

In 2019, a total of 3,921,938, 1,250,665, and 5,863,956 people were included from primary care databases (CPRD GOLD, IPCI, and SIDIAP, respectively); 8,679,416 and 418,743 people were included from primary and outpatient specialist care (IQVIA DA Germany and IQVIA LPD Belgium, respectively); 514,972 from the University Hospital Bordeaux; and 207,016 from the Estonian Biobank.

### Population-level opioid use

A high prevalence of opioid use was observed in primary care, with 12.9% [95% CI: 12.9–13.0] (CPRD GOLD), 9.2% [9.2–9.2] (SIDIAP), and 8.1% [8.0–8.1] (IPCI) of individuals having received opioid prescriptions in 2019 (the year before the COVID-19 pandemic). Prevalence in databases that included inpatient or specialist care varied across settings and geographies, with high opioid prevalence observed in IQVIA LPD Belgium [13.8% (13.7–13.9)] and substantially lower rates in the Estonian Biobank [6.8% (6.7–7.0)], CHUBX [3.6% (3.6–3.7)], and IQVIA DA Germany [3.3% (3.3–3.3)]. In the same year, rates of new opioid prescriptions ranged from 10.0 [10.0–10.1] per 100 person-years in IQVIA LPD Belgium to 2.9 [2.9–2.9] in IQVIA DA Germany.

**FIGURE 1:**
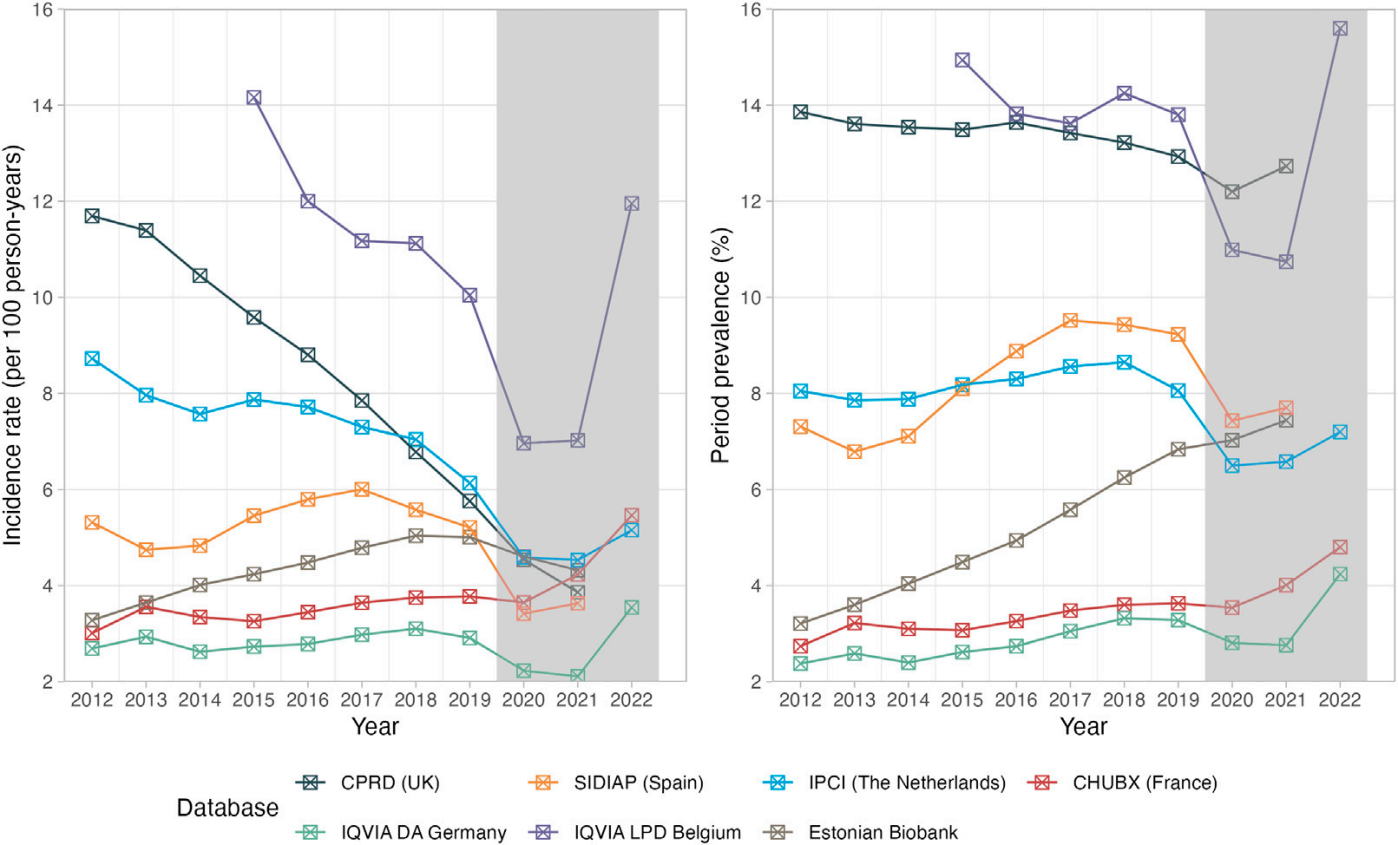
Trends in the incidence and prevalence of opioid prescriptions (2012–2022). Incidence rates are reported as IR/100 person-years. Period prevalence is reported in %. The years 2020–2022 shaded in grey were not included for the interpretation of prescription trends. CPRD GOLD, Clinical Practice Research Datalink GOLD; IPCI, Integrated Primary Care Information Project; SIDIAP, Sistema d’Informació per al Desenvolupament de la Investigació en Atenció Primària; LPD, Longitudinal Patient Database; DA, Disease Analyzer; CHUBX, Clinical Data Warehouse of Bordeaux University Hospital.

Overall, the prevalence of opioid use remained high over the last decade, while a decrease in incidence was observed in many European countries (Figure 1). Between 2012 and 2019, the prevalence of any opioid prescriptions slightly decreased in IQVIA LPD Belgium and CPRD GOLD (−7.6%, and −6.7%, respectively), remained fairly stable in IPCI, and increased by between 26·3% (SIDIAP) and 113·1% (EBB) in all other databases. Notably, the use of prescribed opioids was consistently lower in 2020 and 2021 than in 2019, likely related to disruptions due to the COVID-19 pandemic.

**FIGURE 2:**
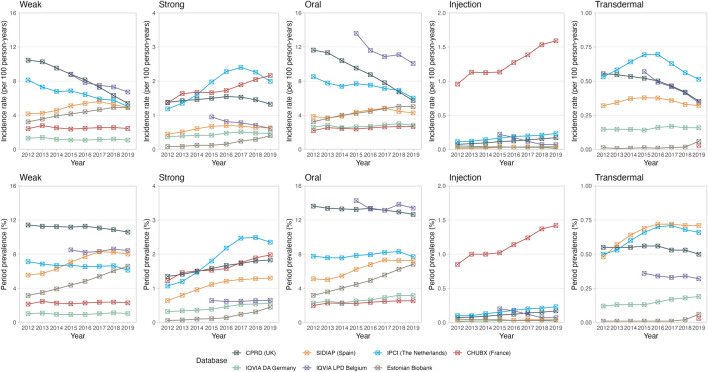
Trends in opioid prescriptions over time, stratified by potency and route of administration. The upper panel represents the incidence rates reported as 100 person-years. The lower panel represents the period prevalences reported as percentage. CPRD GOLD, Clinical Practice Research Datalink GOLD; IPCI, Integrated Primary Care Information Project; SIDIAP, Sistema d’Informació per al Desenvolupament de la Investigació en Atenció Primària; LPD, Longitudinal Patient Database; DA, Disease Analyzer; CHUBX, Clinical Data Warehouse of Bordeaux University Hospital.

Oral formulations contributed the majority of opioid prescriptions, and their prevalence and incidence trends were similar to the overall trend (Figure 2). Opioid injections were predominantly prescribed in the hospital setting (CHUBX) and showed an increasing trend over time while remaining largely stable across all other databases. Trends in the prescription of transdermal opioids appeared to be stable across all databases.

**FIGURE 3:**
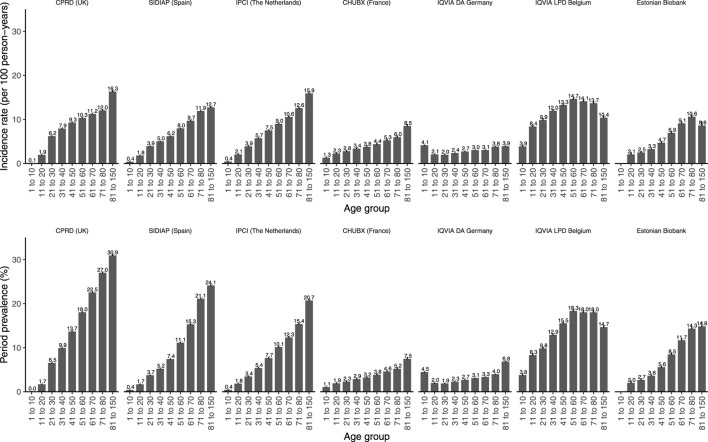
Prevalence and incidence of opioid prescriptions in 2019 stratified by age. The upper panel represents the incidence rates reported as 100 person-years. The lower panel represents the period prevalences reported as percentage. CPRD GOLD, Clinical Practice Research Datalink GOLD; IPCI, Integrated Primary Care Information Project; SIDIAP, Sistema d’Informació per al Desenvolupament de la Investigació en Atenció Primària; LPD, Longitudinal Patient Database; DA, Disease Analyzer; CHUBX, Clinical Data Warehouse of Bordeaux University Hospital.

Opioid utilization was found to be the highest in older age groups (Figure 3), particularly in primary care, with, for example, a very low prevalence of 0.1% [0.1–0.1] being observed in CPRD GOLD in children ≤ 10 years old compared to 30.9% [30.7–31.1] in people aged 81 years and older.

**FIGURE 4:**
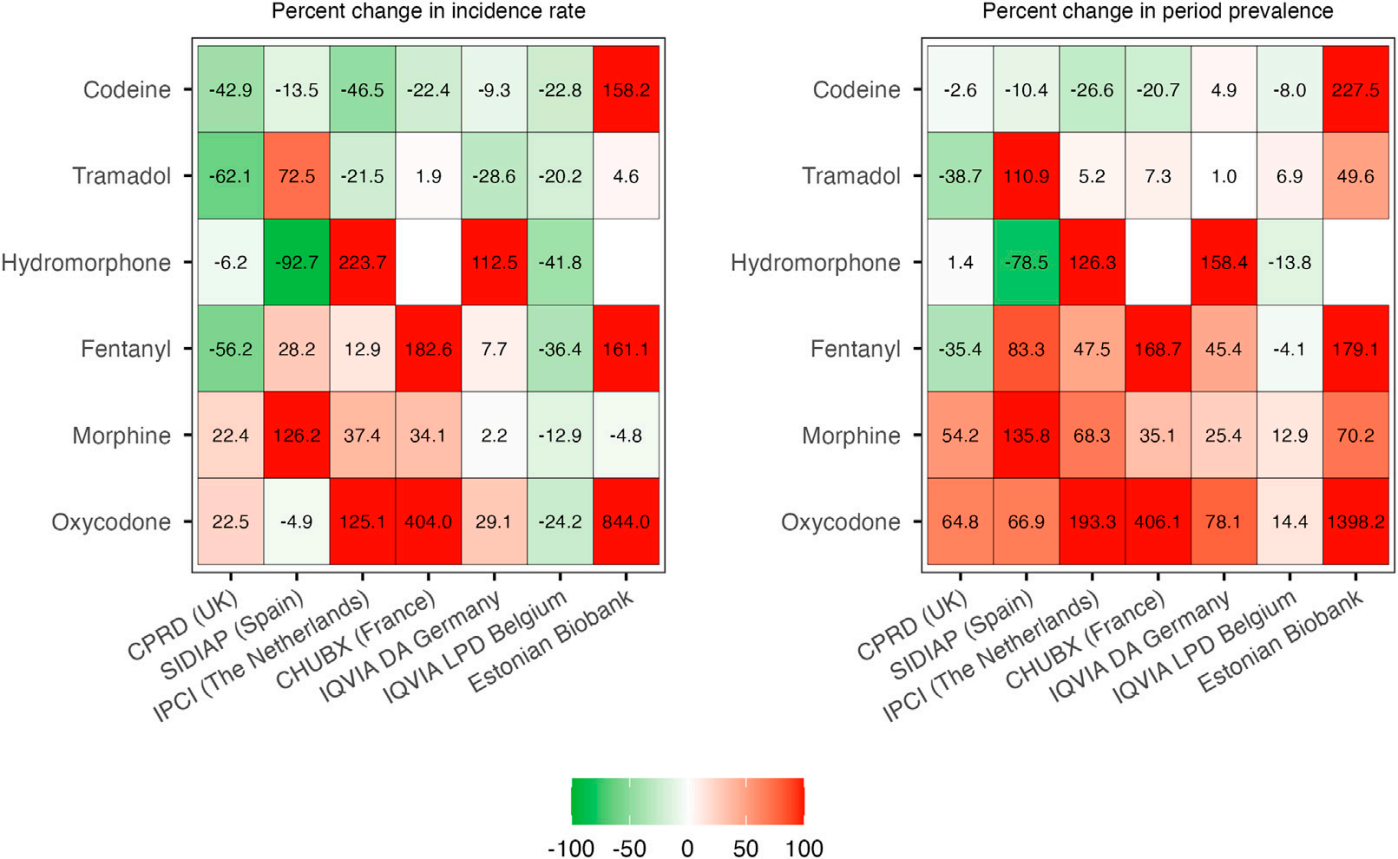
Percent change in individual opioids of special interest: 2019 vs 2012. CPRD GOLD, Clinical Practice Research Datalink GOLD; IPCI, Integrated Primary Care Information Project; SIDIAP, Sistema d’Informació per al Desenvolupament de la Investigació en Atenció Primària; LPD, Longitudinal Patient Database; DA, Disease Analyzer; CHUBX, Clinical Data Warehouse of Bordeaux University Hospital.

Prescription trends for opioid substances of particular interest, including codeine, tramadol, oxycodone, morphine, fentanyl, and hydromorphone, are illustrated as percent change in 2019 vs 2012 (Figure 4) and incidence/prevalence over time in Supplementary Figures S1–S2. New prescriptions of codeine and tramadol decreased in most settings, particularly in CPRD GOLD, IPCI, and IQVIA LPD Belgium, in which high incidences were observed in 2012. Likewise, the incidence of tramadol prescriptions decreased or remained stable, except for primary care data from SIDIAP, where they increased substantially. The prevalence of use of potent opioids such as fentanyl, oxycodone, and morphine increased in most databases (Figure 4; Supplementary Figures S1–S2). Prescription trends (percent change) for all opioids stratified by active substance are provided in Supplementary Figure S3.

### Patient-level opioid use

A total of 6,196,266 new opioid users were included between 2012 and 2022 across all databases, with 4,548,609 (73·4%) included from primary care (CPRD GOLD, SIDIAP, and IPCI), 1,456,142 (23·5%) from primary and outpatient specialist care (IQVIA DA Germany and IQVIA LPD Belgium), 138,171 (2·2%) from a hospital, and 53,344 (0·8%) from a biobank [Table 1]. The median age of new opioid users ranged from 50 to 57 years across all databases, with users of potent opioids usually older than those prescribed weak opioids. More women than men were prescribed opioids in primary care and EBB, whereas no difference was observed in hospital data from CHUBX.

**TABLE 1:** Characteristics of new opioid users in 2012–2022.

Characteristics	CPRD GOLD	SIDIAP	IPCI	IQVIA DA Germany	IQVIA LPD Belgium	CHUBX	Estonian Biobank
N (new opioid users)	2,083,893	2,023,867	440,849	1,248,388	207,754	138,171	53,344
Age	53 [38–69]	55 [40–70]	57 [43–70]	54 [32–70]	50 [34–64]	57 [37–72]	54 [42–65]
Sex, female (%)	1,697,338 (58%)	1,773,685 (59%)	366,540 (60%)	936,414 (57%)	163,545 (56%)	84,635 (51%)	52,659 (69%)
Duration of opioid treatment (overall), median days [IQR]	28 [28–28]	11 [7–31]	10 [7–15]	20 [7–30]	9 [5–25]	2 [1–5]	NA
Proxy for indication*	Backache (5.2%) Low back pain (5%) Pain in the knee region (2.8%)	Common cold (10.9%) Cough (5.4%) Low back pain (3.0%)	Cough (21.5%) Acute upper respiratory tract infection (6.0%) Low back pain (3.7%)	Acute upper respiratory tract infection (15.6%) Cough (14.4%) Nerve root disorder (4.4%)	Cough (26.9%) Common cold (13.7%) Low back pain (10.3%)	Complication of surgical procedure (6.4%) Complication of procedure (6.2%) Headache (5.5%)	Nerve root disorder (11.6%) Pain in spine (9.0%) Cough (8.6%)
N (%), new users of opioids of special interest **							
Codeine	85.53%	53.33%	57.18%	26.86%	37.59%	4.72%	85.53%
Tramadol	21.94%	56.50%	41.84%	14.89%	38.28%	69.72%	21.94%
Hydromorphone	0.03%	0.13%	0.04%	2.28%	0.05%	0.05%	0.03%
Fentanyl	1.80%	7.24%	8.12%	3.89%	2.41%	0.92%	1.80%
Morphine	11.30%	3.37%	7.98%	2.87%	0.69%	46.69%	11.30%
Oxycodone	3.20%	2.04%	24.31%	5.62%	3.11%	18.65%	3.20%
Duration of opioid treatment in days (opioids of special interest)							
Codeine	28 [28–28]	7 [6–11]	10 [8–15]	8 [1–20]	7 [4–20]	3 [1–6]	NA
Tramadol	28 [28–39]	21 [10–61]	10 [8–17]	15 [7–25]	20 [10–34]	2 [1–4]	NA
Hydromorphone	38 [28–82]	81 [31–260]	28 [14–52]	50 [25–76]	30 [15–34]	6 [3–10]	NA
Fentanyl	35 [27–83]	89 [31–225]	23 [14–50]	30 [30–60]	10 [5–30]	4 [1–9]	NA
Morphine	28 [28–54]	33 [11–91]	15 [7–34]	13 [4–30]	15 [7–56]	1 [1–3]	NA
Oxycodone	29 [28–67]	69 [29–175]	12 [6–24]	25 [20–50]	28 [9–30]	3 [1–8]	NA

*Most frequent conditions recorded in 7 days before and on the day of treatment start. **The cohorts of opioids of special interest are not mutually exclusive, and the same person can enter several cohorts. Therefore, the sum of N (new users of opioids of special interest*) for different opioid substances can exceed the counts for N (new opioid users). Summary characteristics are provided as number of individuals N (%) for categorical variables and median [IQR] for continuous covariates. IQR, interquartile range; NA, not available.

Treatment duration varied greatly by setting and active principle: the median duration of any new opioid prescription was 2 days in the CHUBX hospital and ranged from 9 to 20 days for primary care and outpatient specialist care settings. Treatment duration for codeine was shorter than for other opioids in most primary care databases.

The most recorded conditions presenting “proxies” for potential indications comprised respiratory problems (e.g., cough and acute upper respiratory infections) and pain (e.g., back pain) for both primary care and outpatient specialist care databases. Hospital diagnoses included additional indications, like the management of post-operative complications. Cough-related conditions were frequently recorded prior to treatment initiation with codeine, whereas pain was frequently recorded before the initiation of tramadol and potent opioids, such as hydromorphone, fentanyl, morphine, and oxycodone (Supplementary Table S3).

## Discussion

### Key results

This study included more than 20 million participants and 6 million new opioid users across different healthcare settings in seven European countries. The prevalence of opioid use remained high over the last decade, despite a decrease in new opioid prescriptions in many European countries. Of note, the EBB, being the only database with linked claims data, experienced one of the sharpest relative increases in both the prevalence and incidence of opioid use over the study period, despite starting from a lower prevalence. Opioid use was highest among older adults, and the prevalence of potent opioids, such as oxycodone, morphine, and fentanyl, increased during the study period. Treatment duration varied greatly by setting and opioid substance, ranging from few days in the inpatient setting to weeks or months in primary care.

### Results in context

Our findings are in line with previous multinational studies, indicating an increasing trend in opioid use in many European countries. The European Pain Federation conducted an extensive survey on trends of opioid prescriptions and proxies of opioid-related harms with representatives of their national chapters, reporting an increase in opioid prescriptions from 2004 to 2016 in most countries (Hauser et al., 2021). In the United Kingdom, opioid prescriptions increased by 172% from 2004 to 2016 but remained stable from 2016 to 2018 (9). Likewise, another study showed a 216% increase in the prescription of opioids (buprenorphine, fentanyl, morphine, and oxycodone) from 2008 to 2018 (Pierce et al., 2021). Although our study found a similar increase in the prevalence of potent opioids like oxycodone and morphine in the United Kingdom, the prevalence of opioid utilization overall slightly decreased (6·7%). However, two factors complicate direct comparisons between our findings and earlier studies. First, our study used 2012 as the reference year, thereby capturing more recent trends than previous studies. Second, we used the number of opioid users as the metric for quantifying total opioid consumption, which is different from the prior studies using oral morphine equivalents (OMEs). Empirical studies have shown that OME, which converts opioid doses to an equianalgesic dose of oral morphine, can significantly influence study outcomes (Svendsen et al., 2011). For instance, a study based on primary care data in England reported a 34% increase in opioid prescriptions when measured using the prescribed item count, corresponding to a 127% increase when assessed through OME over the same period (Curtis et al., 2019). In contrast to the stable opioid prevalence trends observed in primary care data from the United Kingdom and Belgium, our study showed a notable decrease in the incidence of new prescriptions of over 50% and 29%, respectively, for these two countries. This difference in trends between incidence and prevalence was previously observed in countries like the United States and Australia (Mosher et al., 2015; Lalic et al., 2019) and may reflect a saturation phenomenon in epidemiology, with new opioid users potentially becoming persistent users over the years. However, our study suggested that the duration of opioid treatment was short in most databases but varied logically by setting. For example, a median of 2 days in the French hospital database (CHUBX) likely reflects acute post-operative pain management, while durations of several weeks in primary care databases may represent repeat prescribing patterns or reflect persistent painful conditions. Of note, treatment duration for potent opioids potentially used in palliative care or treatment of chronic cancer pain was, however, longer, particularly in the outpatient setting.

Previous studies indicate that growing numbers of prescriptions of potent opioids, especially fentanyl, morphine, and oxycodone, are of particular concern, given their potential for overdoses, development of addiction, and increased rates of opioid-related deaths involving fentanyl (Gomes et al., 2018). Our findings generally confirmed this. Similar to our study describing the increased use of hydromorphone, the same trend has been reported among long-term care residents in Canada (Iaboni et al., 2019). Furthermore, an increased prevalence of commonly used weak opioids, such as tramadol, has been observed across most countries, with a notable increase in Catalonia, aligning with previous data (Xie et al., 2022; Hurtado et al., 2020). Recent post-marketing studies have raised concerns about the potential safety risk of tramadol (Zeng et al., 2019; Xie et al., 2021). Therefore, its increased prescription warrants close monitoring of the underlying drivers.

The European Pain Federation’s survey reported opioids to be mainly prescribed for acute pain and chronic non-cancer pain in Western and Northern European countries (Hauser et al., 2021). However, our study additionally found substantial use for cough and upper respiratory infections in the outpatient setting, supporting that both pain and respiratory conditions were the most common opioid indications, which is in line with current clinical guidelines. Most recently, to support safe opioid prescribing practices, many countries have provided further recommendations for acute and long-term pain management (Centers for Disease Control and Prevention, 2025; Dowell et al., 2016) and pain relief in palliative care (National Institute for Health and Care Excellence, 2016). Unfortunately, our study did not assess the appropriateness of each prescription, including dosing, duration, and co-medication related to the indication, and therefore cannot conclude whether an opioid use episode was “off-label.”

In 2019, more than 1 in 10 people aged 50 years and older received opioid prescriptions in the outpatient setting (except IQVIA DA Germany). In comparison, a large United States study found that a quarter of Medicare beneficiaries aged 65 years and older used opioids (Jeffery et al., 2018), a figure only slightly higher than those observed in the United Kingdom, Spain, and Belgium databases. Given that advanced age is a well-established risk factor for increased susceptibility to opioid side effects, it is important for future research to determine whether the transition to more persistent use in older adults reflects more effective chronic pain management, improved palliative care, or inadequate prescribing practices in this population.

During the early COVID-19 pandemic, a substantial reduction in drug dispensations was reported in some European countries, including antidiabetics, antihypertensives, antidepressants, and drugs for respiratory diseases (Marengoni et al., 2023; Fornari et al., 2021). In particular, a recent international study analyzed retail pharmacy opioid sales from 66 countries and suggested that the COVID-19 pandemic led to disruptions in opioid purchasing worldwide (Gomes et al., 2022). Our study shows that opioid use was lower than expected across our database populations in 2020 and 2021, corroborating prior preliminary findings. The upward trend in incidence and prevalence from 2021 to 2022 may indicate a return to pre-pandemic levels of healthcare utilization and prescribing, which warrants further study.

### Strengths and limitations

We consider the large study size, including more than 6 million people across Europe with opioid prescriptions, to be a major strength of this study. All databases have undergone extensive quality testing while being onboarded in the DARWIN EU^®^ Network and have been used in previous drug utilization studies. Furthermore, the use of the OMOP Common Data Model, along with standardized and well-tested analytics, contributed to minimizing heterogeneity in the definitions and design approaches, thus facilitating meaningful head-to-head comparisons of opioid utilization across countries and healthcare settings. Summary statistics on patient-level characteristics, treatment durations, and potential indications allowed for a more in-depth understanding of opioid utilization in routine clinical practice, complementing insights from previous multinational surveys and studies focused on serious opioid-related harms.

We acknowledge the following limitations: first, opioids are prescribed in various settings, yet the majority of databases in our study did not include linkage across different settings, and hence, prescription records might have been missed. Consequently, our study might have underestimated the incidence and prevalence of opioid use, with the extent of underestimation likely varying by setting and opioid substance. Moreover, as we used prescription records only, actual intake of opioids and treatment durations might be overestimated. Second, the extent to which our findings are generalizable to the national population or across settings beyond the included databases is limited: primary care databases such as CPRD GOLD, SIDIAP, and IPCI—where GPs serve as the gatekeepers of the national healthcare system—have been shown to be demographically representative of the country’s general population, whereas other databases, such as those from individual hospitals, are not. Third, understanding the indications for opioid prescription is crucial to elucidate the reasons for treatment initiation; however, this information was not recorded in our databases. We, therefore, used conditions recorded up to 1 week before or on the day of opioid prescription as a proxy for a potential indication. Finally, our study did not assess the adequacy of opioid prescriptions. Although huge efforts are being made to promote safe and adequate opioid therapy both locally and nationally, the effects of those programs might not be reflected in our study. For instance, while assessing trends of opioid prescriptions, our study did not take into account whether the incidence of conditions requiring treatment with opioids showed similar trends.

### Implications and conclusion

Previous multinational and time-trend comparisons relied on drug utilization studies that primarily used summary statistics from surveys and monitoring systems (Hauser et al., 2021; Seyler et al., 2021) and sales data (Gomes et al., 2022; Jayawardana et al., 2021; Ju et al., 2022) or focused on opioid dependence or severe opioid-related harms (Jayawardana et al., 2021; Ju et al., 2022; Degenhardt et al., 2019; Robert et al., 2023). Instead, our study analyzed millions of individual prescription records, which offered the most granular picture of recent trends in opioid prescriptions and user characteristics in Europe. Despite certain limitations, the foundational findings could have several important implications. First, they establish a robust, evidence-based baseline that regulatory agencies can use to evaluate the impact of policies against a reliable benchmark. Second, they may inform future opioid utilization study focus. For instance, the observed increase in potent opioid use among the elderly generates a clear mandate for targeted research into prescribing appropriateness within this vulnerable population. Finally, and most critically, these findings help prioritize and inform public health interventions. The increasing use of potent opioids, like oxycodone and fentanyl, points to potential public health harms, including heightened risks of dependence and overdose. To mitigate these risks, a multifaceted approach is needed, promoting adherence to evidence-based prescribing guidelines, supporting clinical decision-making for safer alternatives, and implementing robust monitoring systems to track the use of potent opioids.

In summary, despite a decrease in new opioid prescriptions in many European countries, the prevalence of opioid use remained largely stable over the last decade and was particularly high among the elderly population. In addition, the number of people in Europe being prescribed potent opioids such as hydromorphone, oxycodone, and fentanyl has been increasing in recent years. Continuous monitoring of evolving opioid prescription patterns will be helpful in the post-pandemic era, and future studies on the adequacy of opioid prescriptions are critically needed to identify and address inappropriate prescribing.

## Data Availability

The data analyzed in this study are subject to the following licenses/restrictions: CPRD GOLD: CPRD GOLD data were obtained under the CPRD multi-study license held by the University of Oxford after Research Data Governance (RDG) approval. Direct data sharing is not allowed. SIDIAP: In accordance with the current European and national law, the data used in this study are only available to the researchers participating in this study. Thus, the authors are not permitted to distribute the data or make them publicly available to third parties. However, researchers from public institutions can request data from SIDIAP if they comply with certain requirements. Further information is available online (https://www.sidiap.org/index.php/menu-solicitudesen/application-proccedure) or can be obtained by contacting SIDIAP (sidiap@idiapjgol.org). IPCI: Almost all studies using IPCI data concern retrospective research on observational data and, as such, are not subject to the Medical Research Involving Human Subjects Act (WMO) and do not require approval from a medical research ethical committee. If additional patient data have to be collected, the protocol is sent to an accredited medical research ethical committee for review. Before data are transferred to the central database, they are pseudonymized. The data are stored on an isolated network without an internet connection. Within this network, there are separate layers to distinguish access for data supply to GPs, data coding, and data analysis. Data access is only possible on authorized computers located in secure facilities and is restricted to authorized users. Employees working with the IPCI and external researchers must sign a declaration of confidentiality. Only aggregated data are allowed to leave the secure environment. The study protocol was approved by the review board of IPCI on 22 June 2023. IQVIA: IQVIA Germany DA and IQVIA LPD Belgium in the OMOP format are commercially available databases that can be purchased and licensed by any researcher. The collection and de-identification of these data assets is a process that is commercial intellectual property and not privileged to the data licensees and the co-authors of this study. Licensees of these data have signed Data Use Agreements with the data vendors, which detail the usage protocols for running retrospective research on these databases. All analyses performed in this study were in accordance with the Data Use Agreement terms as specified by the data owners. As these data are deemed commercial assets, there is no Institutional Review Board applicable to the usage and dissemination of these result sets or required registration of the protocol with additional ethics oversight. Compliance with Data Use Agreement terms, which stipulate how these data can be used and for what purpose, is sufficient for licensing commercial entities. Further inquiry related to the governance oversight of these assets can be made with the respective commercial entity: IQVIA (https://iqvia.com). At no point in the course of this study were the authors exposed to identified patient-level data. All result sets represent aggregate, de-identified data that are represented at a minimum cell size of 5 to reduce the potential for re-identification. EBB: Access to data from the Estonian Biobank can only be requested directly from the Estonian Biobank (https://genomics.ut.ee/en/content/estonian-biobank) upon approval by its scientific committee and the Estonian Committee on Bioethics and Human Research. CHUBX: In accordance with European and national regulations, no personal data were transmitted as part of this study; only aggregated data resulting from analyses carried out locally were shared. Patients who expressed opposition to participating in the research were removed from the analysis. Participation in this study was declared to and validated by the hospital’s DPO and the IRB.
