# Supplementary material for: Trends in prescription opioid use in Europe: A DARWIN EU® multinational cohort study including seven European countries
Source: Front Pharmacol. 2025 Aug 18;16:1608051. doi: 10.3389/fphar.2025.1608051 (PMC12399544; doi:10.3389/fphar.2025.1608051)
Supplement: Supplementary file 1 [file DataSheet1.docx]

# Supplementary Material

Trends of prescription opioid use in Europe: A DARWIN EU® multinational cohort study including 7 European countries

Supplement Table 1: Detailed description of the respective databases

| Country | Name of Database | Health Care setting | Type of Data | Number of active subjects | Data lock for the last update |
| --- | --- | --- | --- | --- | --- |
| The Netherlands | IPCI | Primary care | EHR | 1.39 million | 1/12/2022 |
| France | CHUBX | Secondary care (in and outpatients) | EHR | 2.13 million | 1/3/2023 |
| Spain | SIDIAP | Primary care | EHR | 5.8 million | 1/6/2022 |
| Belgium | IQVIA LPD Belgium | outpatient specialist care | EHR | 0.4 million | 1/1/2022 |
| Germany | IQVIA DA Germany | outpatient specialist care | EHR | 8.5 million | 1/9/2022 |
| Estonia | EBB | Biobank | Claims data | 0.2 million | 31/3/2021 |
| United Kingdom | CPRD GOLD | Primary Care | EHR | 2.97 million | 31/1/2023 |

Supplement Table 2: Concept sets for opioids included in this study

**Opioids:** Individual substances incl. mono-preparations and combinations

| **Concept name** | **Concept id** | **Including all descendants?** |
| --- | --- | --- |
| Alfentanil | 19059528 | Yes |
| Anileridine | 19032662 | Yes |
| Bezitramide | 37493802 | Yes |
| Buprenorphine | 1133201 | Yes |
| Butorphanol | 1133732 | Yes |
| Codeine | 1201620 | Yes |
| Dextromethorphan | 1119510 | Yes |
| Dextromoramide | 19021940 | Yes |
| Dezocine | 19088393 | Yes |
| Dihydrocodeine | 1189596 | Yes |
| Dimemorfan | 36852751 | Yes |
| Ethylmorphine | 19050414 | Yes |
| Fentanyl | 1154029 | Yes |
| Hydrocodone | 1174888 | Yes |
| Hydromorphone | 1126658 | Yes |
| Ketobemidone | 40798904 | Yes |
| Meperidine | 1102527 | Yes |
| Meptazinol | 19003010 | Yes |
| Methadone | 1103640 | Yes |
| Morphine | 1110410 | Yes |
| Nalbuphine | 1114122 | Yes |
| Nicomorphine | 37493805 | Yes |
| Normethadone | 19015787 | Yes |
| Noscapine | 19021930 | Yes |
| Oliceridine | 37002667 | Yes |
| Opium | 923829 | Yes |
| Oxycodone | 1124957 | Yes |
| Oxymorphone | 1125765 | Yes |
| Papaveretum | 19129648 | Yes |
| Pentazocine | 1130585 | Yes |
| Phenazocine | 19132884 | Yes |
| Phenoperidine | 19132889 | Yes |
| Pholcodine | 19024213 | Yes |
| Pirinitramide | 19134009 | Yes |
| Propoxyphene | 1153664 | Yes |
| Remifentanil | 19016749 | Yes |
| Sufentanil | 19078219 | Yes |
| Tapentadol | 19026459 | Yes |
| Thebacon | 40799139 | Yes |
| Tilidine | 19002431 | Yes |
| Tramadol | 1103314 | Yes |

**Weak and Potent Opioids**

| **Concept name** | **Concept id** | **Including all descendants?** |
| --- | --- | --- |
| **Weak Opioids** |  |  |
| Codeine | 1201620 | Yes |
| Hydrocodone | 1174888 | Yes |
| Tramadol | 1103314 | Yes |
|  |  |  |
| **Potent Opioids** |  |  |
| Buprenorphine | 1133201 | Yes |
| Fentanyl | 1154029 | Yes |
| Hydromorphone | 1126658 | Yes |
| Methadone | 1103640 | Yes |
| Morphine | 1110410 | Yes |
| Oxycodone | 1124957 | Yes |
| Oxymorphone | 1125765 | Yes |
| Tapentadol | 19026459 | Yes |

**Opioids by route of administration**

| **Route of administration** | **Dose Form** | **Including all descendants?** |
| --- | --- | --- |
| oral | Oral Granules, Sublingual Tablet, Oral Pellet, Chewable Extended Release Oral Tablet, Oral Flakes, Oral Gel, Oral Foam, Extended Release Oral Capsule, Oral Paste, Oral Strip, Extended Release Oral Tablet, Delayed Release Oral Tablet, Oral Cream, Powder for Oral Suspension, Oral Spray, Chewable Tablet, Granules for Oral Solution, Powder for Oral Solution, Oral Lozenge,  Buccal Tablet, Oral Granules (Effervescents), Granules for Oral Suspension, Oral Solution, Chewing Gum, Oral Ointment, Oral Wafer, Oral Powder, Oral Suspension, Sustained Release Buccal Tablet, Disintegrating Oral Tablet, Effervescent Oral Tablet, Oral Capsule, Delayed Release Oral Capsule, Oral Tablet, Sublingual Powder, Delayed Release Oral Granules, Tablet for Oral Suspension, Oral Film, Sublingual Film, Buccal film | Yes |
| transdermal | Transdermal System, Medicated Patch | Yes |
| Parenteral (injection) | Auto-Injector, Prefilled Applicator, Intravenous Solution, Prefilled Syringe, Soft Tissue Injection Suspension, Intrathecal Suspension, Intraperitoneal Solution, Intratracheal Suspension, Extended Release Suspension, Intramuscular Solution, Pen Injector, Injection, Irrigation Solution, Intravenous Suspension, Injectable Foam, Intramuscular Prolonged Release Suspension, Injectable Suspension, Injectable Solution, Jet Injector | Yes |

Supplement Figure 1 Trajectory of prevalent opioid prescriptions of special interest from 2012 to 2019


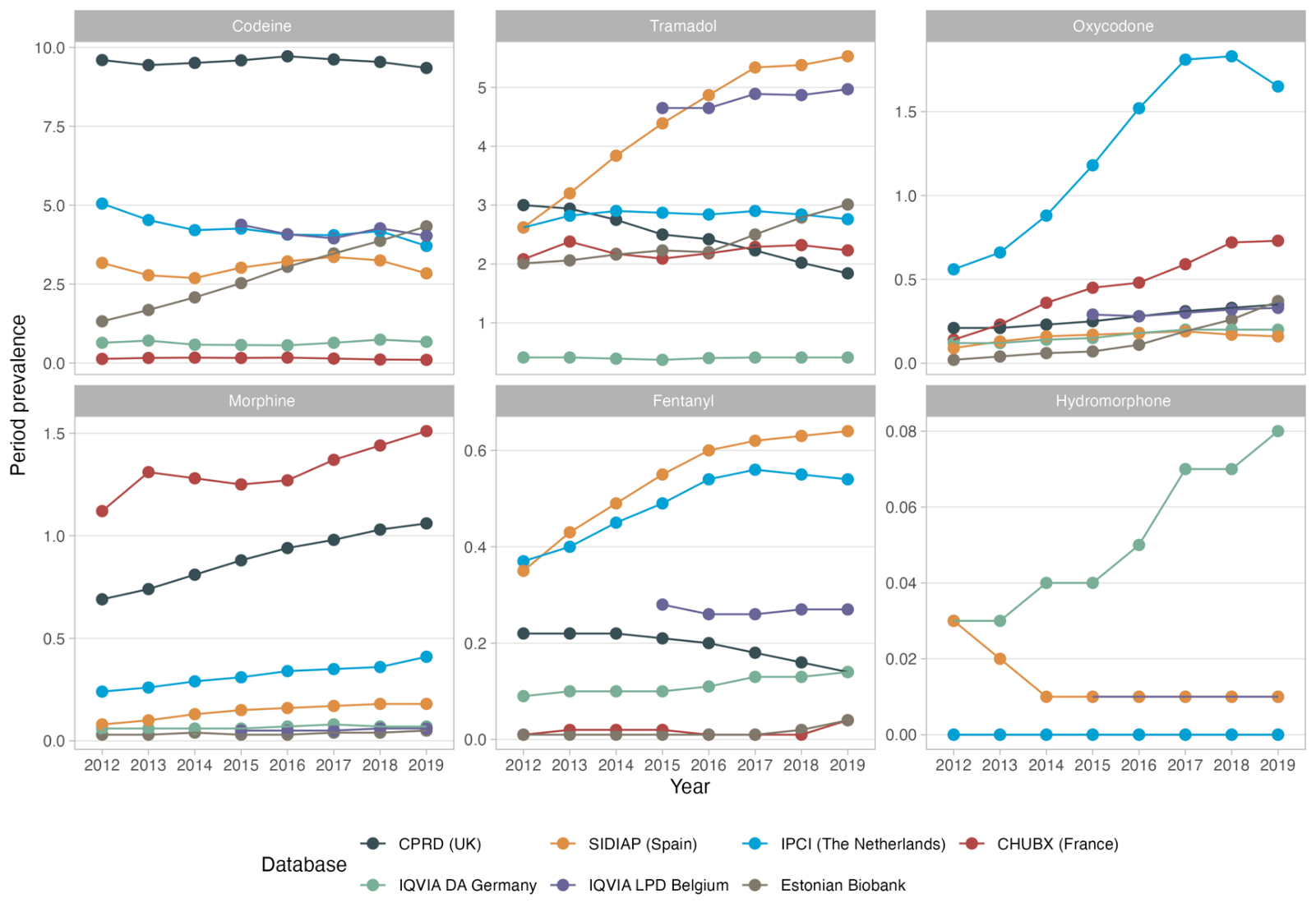


Period prevalence is reported in %. CPRD GOLD = Clinical Practice Research Datalink GOLD, IPCI = Integrated Primary Care Information Project, SIDIAP = Sistema d’Informació per al Desenvolupament de la Investigació en Atenció Primària, LPD = Longitudinal Patient Database, DA = Disease Analyzer, CHUBX = Clinical Data Warehouse of Bordeaux University Hospital (CHUBX)

Supplement Figure 2: Trajectory of incident opioid prescriptions of special interest from 2012 to 2019.


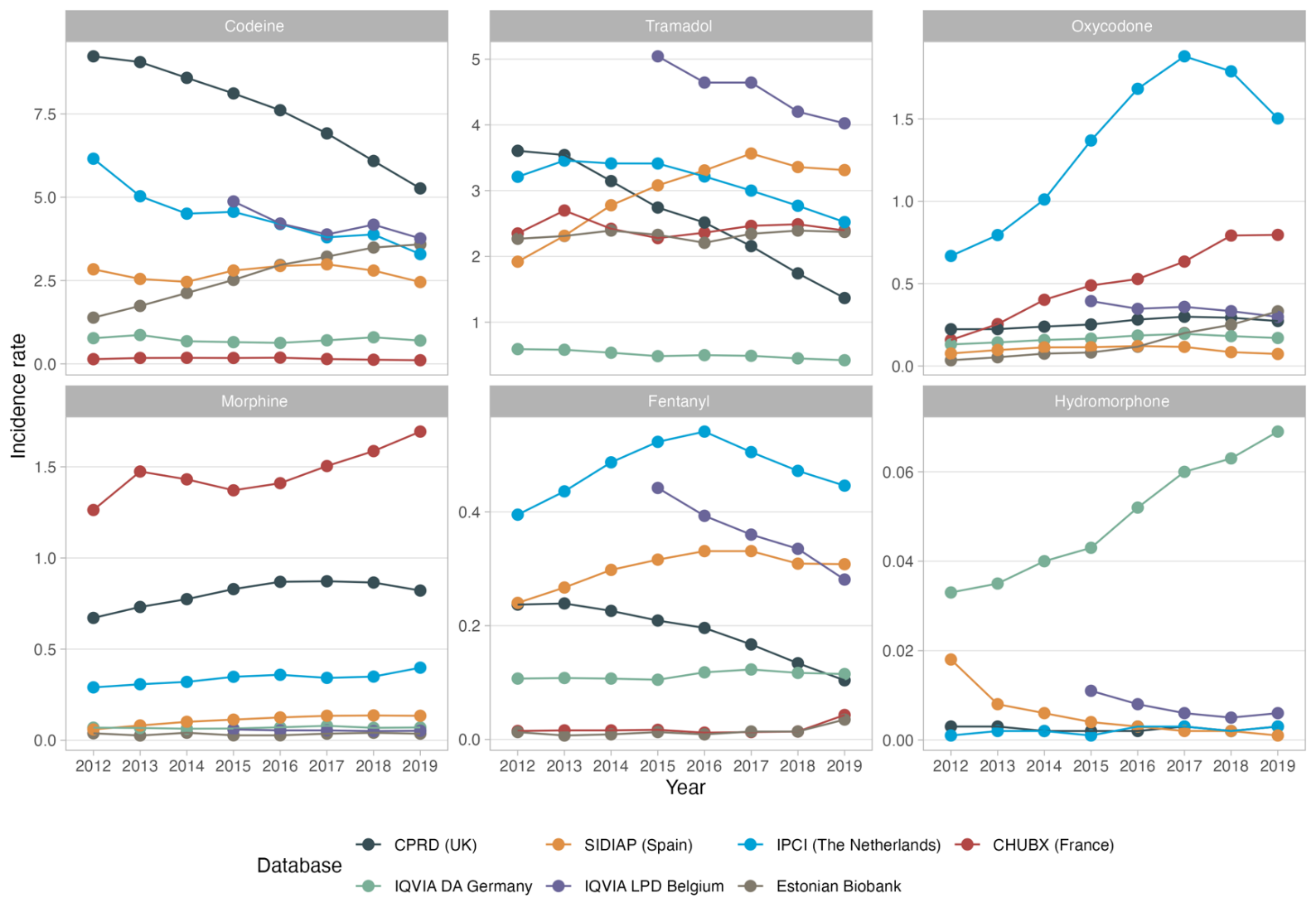


Incidence rates are reported as IR/100 person years. CPRD GOLD = Clinical Practice Research Datalink GOLD, IPCI = Integrated Primary Care Information Project, SIDIAP = Sistema d’Informació per al Desenvolupament de la Investigació en Atenció Primària, LPD = Longitudinal Patient Database, DA = Disease Analyzer, CHUBX = Clinical Data Warehouse of Bordeaux University Hospital (CHUBX)

Supplement Figure 3: Percent change of individual opioid substances 2012 to 2019.


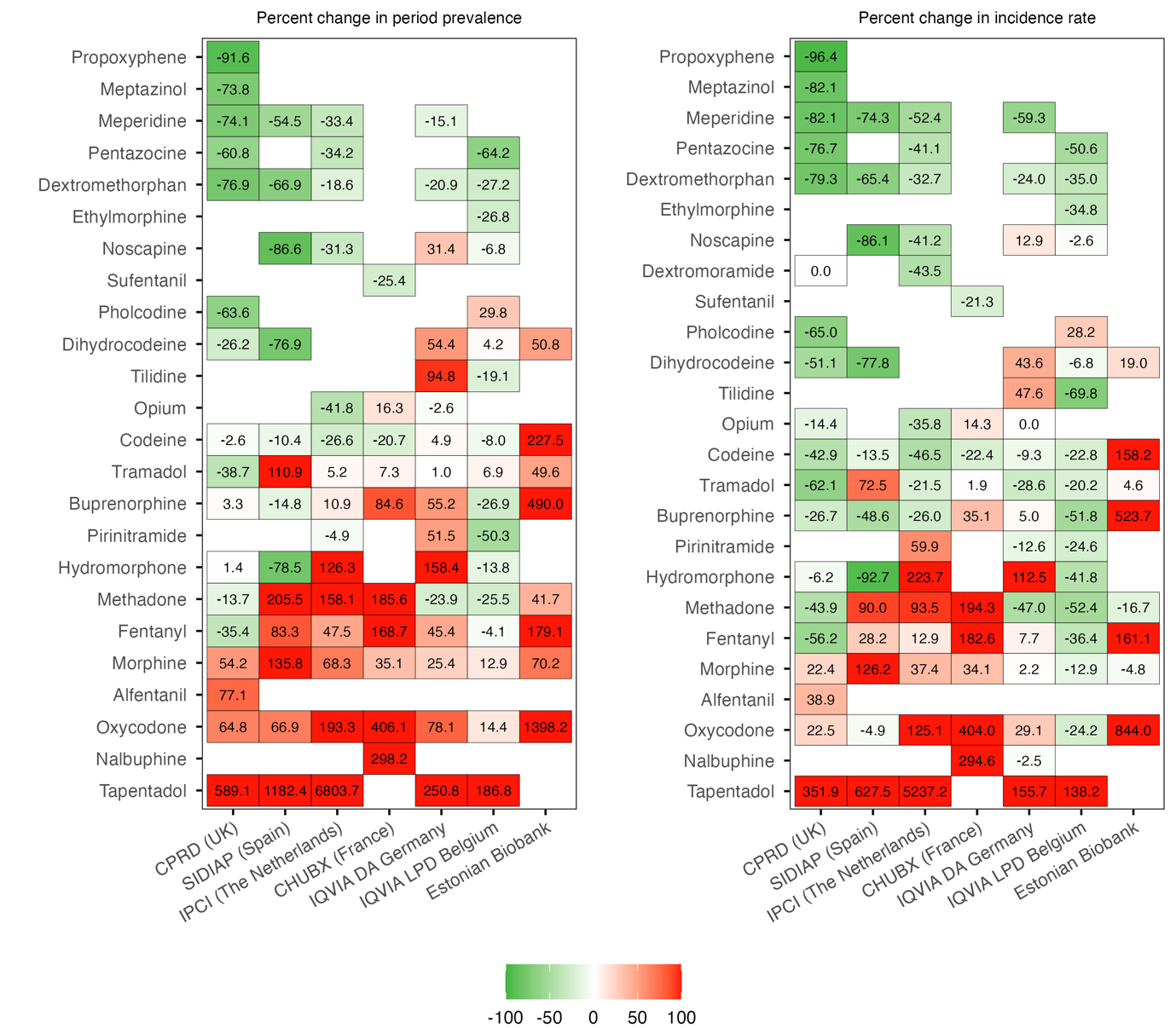


CPRD GOLD = Clinical Practice Research Datalink GOLD, IPCI = Integrated Primary Care Information Project, SIDIAP = Sistema d’Informació per al Desenvolupament de la Investigació en Atenció Primària (SIDISP), LPD = Longitudinal Patient Database, DA = Disease Analyzer, CHUBX = Clinical Data Warehouse of Bordeaux University Hospital (CHUBX)

Supplement Table 3: Proxy for indication based on condition recorded within 7 days from incident opioid prescription, stratified for opioid substances of special interest

|  | **CPRD GOLD** | | **SIDIAP** | | **IPCI** | | **IQVIA DA Germany** | | **IQVIA LPD Belgium** | | **CHUBX** | | **Estonian Biobank** | |
| --- | --- | --- | --- | --- | --- | --- | --- | --- | --- | --- | --- | --- | --- | --- |
| Codeine | Backache | 5·3 | Common cold | 21·2 | Cough | 40·9 | Acute upper respiratory infection | 21·8 | Cough | 29·2 | Headache | 6·9 | Cough | 13·6 |
|  | Low back pain | 5·1 | Cough | 10·1 | Acute upper respiratory infection | 10·9 | Cough | 20·3 | Common cold | 15·6 | Acquired absence of organ | 6·4 | Nerve root disorder | 9·6 |
|  | Pain of knee region | 3·0 | Acute pharyngitis | 5·1 | COVID-19 | 2·0 | Acute pharyngitis | 2·7 | Acute upper respiratory infection | 9·7 | Complication of procedure | 5·5 | Pain in spine | 7·4 |
|  | Shoulder pain  Neck pain | 2·6  1·9 | Acute lower respiratory tract infection | 2·3 | Common cold | 1·6 | Common cold | 2·2 | Acute tracheitis | 8·5 | Complication of surgical procedure | 4·8 | Acute upper respiratory infection | 5·8 |
|  | Cough | 1·8 | Acute upper respiratory infection | 1·4 | Pneumonia | 1·5 | Nerve root disorder | 1·9 | Acute sinusitis | 7·8 | Coronary artery graft present | 4·8 | Low back pain | 5·4 |
| Tramadol | Backache | 5·4 | Low back pain | 5·6 | Low back pain | 8·6 | Nerve root disorder | 11·9 | Low back pain | 28·6 | Complication of surgical procedure | 7·9 | Nerve root disorder | 17·4 |
|  | Low back pain | 5·3 | Joint pain | 1·8 | Finding of back | 7·4 | Low back pain | 7·2 | Pain | 13·9 | Complication of procedure | 7·3 | Pain in spine | 11·7 |
|  | Pain of knee region | 2·8 | Lumbago with sciatica | 1·6 | Backache with radiation | 7·1 | Lumbago with sciatica | 6·8 | Lumbago with sciatica | 6·6 | Headache | 5·7 | Intervertebral disc disorder | 9·7 |
|  | Shoulder pain | 2·3 | Neck pain | 1·5 | Finding of shoulder region | 4·5 | Chronic pain | 4·1 | Spondylosis | 5·8 | Acquired absence of organ | 5·1 | Low back pain | 8·0 |
|  | Hip pain | 1·9 | Traumatic AND/OR non-traumatic injury | 1·5 | Finding of neck region | 2·9 | Intervertebral disc prolapse | 4·0 | Esophagitis | 5·1 | Coronary artery graft present | 4·7 | Osteoarthritis of knee | 5·4 |

|  | **CPRD GOLD** | | **SIDIAP** | | **IPCI** | | **IQVIA DA Germany** | | **IQVIA LPD Belgium** | | **CHUBX** | | **Estonian Biobank** | |
| --- | --- | --- | --- | --- | --- | --- | --- | --- | --- | --- | --- | --- | --- | --- |
| Hydromorphone | Pain | 1·9 | Low back pain | 1·1 | Malignant tumor of lung | 5·4 | Chronic pain | 11·6 | Pain | 29·0 |  |  |  |  |
|  |  |  | Chronic pain | 0·7 | Localized abdominal pain | 2·7 | Nerve root disorder | 3·7 | Low back pain | 17·8 |  |  |  |  |
|  |  |  | Osteoarthritis | 0·5 | Malignant tumor of urinary bladder | 2·7 | Pain | 3·3 | Polyneuropathy | 8·4 |  |  |  |  |
|  |  |  | Joint pain | 0·4 |  |  | Chronic intractable pain | 2·2 | Backache | 4·7 |  |  |  |  |
|  |  |  | Traumatic AND/OR non-traumatic injury | 0·4 |  |  | Primary malignant neoplasm of resp. tract | 2·1 | Pain in limb | 4·7 |  |  |  |  |
| Fentanyl | Pain | 3·1 | Low back pain | 1·7 | Finding of back | 4·9 | Chronic pain | 13·5 | Low back pain | 20·4 | Secondary malignant neoplasm of bone | 13·5 | Chronic intractable pain | 10·6 |
|  | Backache | 2·9 | Chronic pain | 1·5 | Low back pain | 4·2 | Pain | 4·5 | Pain | 19·8 | Anemia in neoplastic disease | 12·3 | Primary malignant neoplasm of prostate | 5·9 |
|  | Low back pain | 2·0 | Traumatic AND/OR non-traumatic injury | 1·5 | Malignant tumor of lung | 4·1 | Nerve root disorder | 3·2 | Polyarthropathy | 6·1 | Chronic pain | 11·3 | Primary malignant neoplasm of head of pancreas | 5·3 |
|  | Hip pain | 1·1 | Joint pain | 0·9 | Backache with radiation | 3·5 | Chronic intractable pain | 2·6 | Backache | 4·8 | Acquired absence of organ | 11·1 | Anemia in neoplastic disease | 5·3 |
|  | Pain of knee region | 1·0 | Primary malignant neoplasm of respiratory tract | 0·8 | Generalized aches and pains | 2·6 | Low back pain | 1·4 | Neuralgia | 3·8 | Abdominal pain | 10·9 | Primary malignant neoplasm of lung | 4·7 |
| Morphine | Backache | 2·7 | Traumatic AND/OR non-traumatic injury | 2·7 | Urolithiasis | 5·4 | Chronic pain | 7·7 | Pain | 25·5 | Complication of surgical procedure | 7·1 | Chronic intractable pain | 9·7 |
|  | Low back pain | 2·1 | Primary malignant neoplasm of resp. tract | 1·5 | Localized abdominal pain | 4·3 | Inflammatory disorder of digestive tract | 4·0 | Low back pain | 13·5 | Complication of procedure | 6·9 | Anemia in neoplastic disease | 5·8 |
|  | Pain | 2·0 | Acute lower respiratory tract infection | 1·3 | Backache with radiation | 4·3 | Pain | 3·6 | Pain in limb | 3·3 | Acquired absence of organ | 5·5 | Primary malignant neoplasm of lung | 5·3 |
|  | Abdominal pain | 1·6 | Urinary tract infectious disease | 1·1 | Malignant tumor of lung | 4·1 | Nerve root disorder | 2·4 | Primary malignant neoplasm of resp. tract | 3·3 | Abdominal pain | 5·0 | Primary malignant neoplasm of prostate | 5·1 |
|  | Pain of knee region | 1·1 | Chronic pain | 0·7 | Finding of back | 3·9 | Primary malignant neoplasm of resp. tract | 2·1 | Spondylosis | 3·2 | Acute pain | 4·9 | Multiple myeloma | 4·8 |
| Oxycodone | Backache | 1·7 | Low back pain | 1·7 | Backache with radiation | 5·9 | Chronic pain | 10·1 | Pain | 20·7 | Complication of surgical procedure | 11·7 | Chronic intractable pain | 12·7 |
|  | Pain | 1·7 | Traumatic AND/OR non-traumatic injury | 1·0 | Finding of back | 4·9 | Nerve root disorder | 6·3 | Low back pain | 20·6 | Complication of procedure | 9·8 | Nerve root disorder | 9·6 |
|  | Low back pain | 1·2 | Chronic pain | 0·9 | Low back pain | 4·5 | Intervertebral disc prolapse | 3·8 | Lumbago with sciatica | 7·1 | Acquired absence of organ | 9·0 | Primary gonarthrosis, bilateral | 8·0 |
|  | Abdominal pain | 0·8 | Joint pain | 0·7 | Traumatic injury | 2·6 | Pain | 3·0 | Esophagitis | 6·0 | Abdominal pain | 6·4 | Primary coxarthrosis, bilateral | 7·8 |
|  | Pain of knee region | 0·8 | Lumbago with sciatica | 0·7 | Finding of shoulder region | 2·3 | Spinal stenosis | 3·0 | Spondylosis | 5·9 | Headache | 6·3 | Intervertebral disc disorder | 6·7 |

Supplement Table 3 continued
